# Antiplasmodial activity of chloroquine analogs against chloroquine-resistant parasites, docking studies and mechanisms of drug action

**DOI:** 10.1186/1475-2875-13-469

**Published:** 2014-12-02

**Authors:** Nicolli B de Souza, Arturene ML Carmo, Adilson D da Silva, Tanos CC França, Antoniana U Krettli

**Affiliations:** Centro de Pesquisas René Rachou, FIOCRUZ Minas, Av. Augusto de Lima 1715, Belo Horizonte, 30190-002 MG Brazil; Departamento de Química, Instituto de Ciências Exatas, Universidade Federal de Juiz de Fora, Rua José Lourenço Kelmer s/n, Juiz de Fora, 36036-900 MG Brazil; Laboratório de Modelagem Molecular Aplicada à Defesa Química e Biológica, Instituto Militar de Engenharia, Praça General Tibúrcio 80, Rio de Janeiro, 22290-270 RJ Brazil

**Keywords:** Malaria, Chloroquine analogs, Diaminealkyne, Diaminedialkyne, *P. falciparum*, Lactate-dehydrogenase enzyme, Docking

## Abstract

**Background:**

Given the threat of resistance of human malaria parasites, including to artemisinin derivatives, new agents are needed. Chloroquine (CQ) has been the most widely used anti-malarial, and new analogs (CQAns) presenting alkynes and side chain variations with high antiplasmodial activity were evaluated.

**Methods:**

Six diaminealkyne and diaminedialkyne CQAns were evaluated against CQ-resistant (CQ-R) (W2) and CQ-sensitive (CQ-S) (3D7) *Plasmodium falciparum* parasites in culture. Drug cytotoxicity to a human hepatoma cell line (HepG2) evaluated, allowed to calculate the drug selectivity index (SI), a ratio of drug toxicity to activity *in vitro*. The CQAns were re-evaluated against CQ-resistant and -sensitive *P. berghei* parasites in mice using the suppressive test. Docking studies with the CQAns and the human (*Hss*LDH) or plasmodial lactate dehydrogenase (*Pf*LDH) enzymes, and, a β-haematin formation assay were performed using a lipid as a catalyst to promote crystallization *in vitro.*

**Results:**

All tested CQAns were highly active against CQ-R *P. falciparum* parasites, exhibiting half-maximal inhibitory concentration (IC_50_) values below 1 μΜ. CQAn33 and CQAn37 had the highest SIs. Docking studies revealed the best conformation of CQAn33 inside the binding pocket of *Pf*LDH; specificity between the residues involved in H-bonds of the *Pf*LDH with CQAn37. CQAn33 and CQAn37 were also shown to be weak inhibitors of *Pf*LDH. CQAn33 and CQAn37 inhibited β-haematin formation with either a similar or a 2-fold higher IC_50_ value, respectively, compared with CQ. CQAn37 was active in mice with *P. berghei*, reducing parasitaemia by 100%. CQAn33, -39 and -45 also inhibited CQ-resistant *P. berghei* parasites in mice, whereas high doses of CQ were inactive.

**Conclusions:**

The presence of an alkyne group and the size of the side chain affected anti-*P. falciparum* activity *in vitro*. Docking studies suggested a mechanism of action other than *Pf*LDH inhibition. The β-haematin assay suggested the presence of an additional mechanism of action of CQAn33 and CQAn37. Tests with CQAn34, CQAn37, CQAn39 and CQAn45 confirmed previous results against *P. berghei* malaria in mice, and CQAn33, 39 and 45 were active against CQ-resistant parasites, but CQAn28 and CQAn34 were not. The result likely reflects structure-activity relationships related to the resistant phenotype.

## Background

Malaria remains a major public health problem, resulting in 207 million cases and 627,000 deaths worldwide in 2012 [1]. There is no available vaccine, and the control of the disease relies on the use of bed nets, other individual protection against mosquito bites, and the successful drug treatment of infected patients [2]. Presently, chemotherapy has been hampered by the low sensitivity of the parasite to most available anti-malarial drugs [3], including artemisinin derivatives [4–6]. Resistance to chloroquine, in the case of *P. falciparum*, is linked to mutations in the *P. falciparum* chloroquine resistance transporter (*pfcrt*) gene, which alters the transport and accumulation of the drug in the digestive vacuole (DV) of the parasite [7, 8]. *Plasmodium vivax* resistance to CQ has been described [9, 10] and seems related to the intense malaria morbidity in the Amazon region [11]. However, neither CQ-R nor mutation markers were detected among *P. vivax* in recent studies of 47 isolates from the West Amazon, all from patients with non-severe malaria [12].

Chemical modifications of CQ have been used as a powerful strategy to find new anti-*P. falciparum* agents effective against drug-resistant parasites [13, 14]. Structural modifications of the alkyldiamine side chains have provided rather active compounds with decreased cross-resistance to CQ [15] and with different structure-activity relationships [16–18]. In the present work, six CQ analogs (CQAns) that reduced parasitaemia in *P. berghei*-infected mice [19], comprising diaminealkynes and diaminedialkynes, were evaluated *in vitro* against CQ-R (W2 clone) and CQ-S (3D7 strain) forms of *P. falciparum* parasites in cultures. These CQAns were then reevaluated in mice with CQ-sensitive or CQ-resistant *P. berghei* parasites. The compounds with the best selectivity index, based on drug cytotoxicity and activity *in vitro*, were evaluated for the inhibition of β-hematin formation. To clarify the possible mechanism of action of the CQAns evaluated herein, docking studies were also performed based on interactions between the CQAns and the plasmodial lactate dehydrogenase (*Pf*LDH) enzyme, a known target of 4-aminoquinolines [20, 21].

## Methods

### Synthesis of the molecules

**Figure 1:**
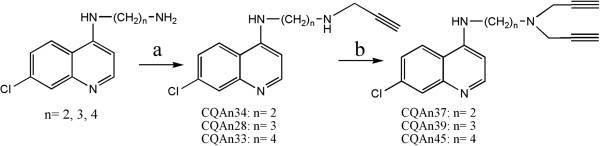
**Scheme of CQAns synthesis. a)** 2 eq. propargyl bromide, K_2_CO_3_, EtOH, 0°C, 72 h, yield: 50 to 60%; **b)** 4 eq. propargyl bromide, K_2_CO_3_, EtOH, 0°C, 72 h, yield: 50 to 60%.

Drug synthesis was performed as previously reported by Da Silva *et al*. [22]. In brief, 4-alkyldiamino-7-chloroquinolines were treated with 2 eq. of propargyl bromide and K_2_CO_3_ in EtOH at 0°C for 72 h to produce the compounds CQAn28, CQAn33, and CQAn34 at a 50% to 60% yield. The addition of 4 eq. of propargyl bromide under the same conditions led to the production of the compounds CQAn37, CQAn39, and CQAn45 at a 50% to 60% yield (Figure 1). All the compounds were characterized via one-dimensional nuclear magnetic resonance (1D-NMR), infrared (IR) spectroscopy, and melting point (MP) assays, and the results were in accordance with data in the literature [22].

### Cytotoxicity tests *in vitro*

A human hepatoma cell line (HepG2), originally received from the University of Lisbon as a kind gift, was cultured as described [23]. The cells were maintained in RPMI 1640 medium (Sigma-Aldrich, ref 6504) supplemented with 40 mg/L gentamicin and 10% heat-inactivated foetal calf serum (FCS), and they were maintained in a 5% CO_2_ atmosphere at 37°C. When confluent, the cell monolayer was trypsinized, washed with culture medium supplemented with 10% FCS, counted, diluted (5 × 10^3^ cells/well), placed in flat-bottom 96-well plates (Corning, Santa Clara, CA, USA), and incubated for 18 h at 37°C to allow cell adhesion. The compounds were added to the plates at concentrations up to 1,700 μM, followed by incubation for an additional 24 h, after which 20 μL (5 μg/mL) of 3-(4,5-dimethylthiazol-2-yl)-2,5 diphenyltetrazolium bromide (MTT) solution was added per well to evaluate drug cytotoxicity. After 3 h, the supernatant was discarded, 100 μL of dimethyl sulphoxide (DMSO) was added per well, and the optical density was measured (SpectraMax340PC384, Molecular Devices) at 570 nm (test) and 630 nm (background). Cell viability was expressed as the percentage of the absorbance compared to the absorbance of the untreated cells and subtracted from the appropriate background measurement. The lethal drug dose for 50% of the cells (MLD_50_) was determined as described [24] and was used to calculate the SI of the active compounds, a ratio of the *in vitro* cell toxicity and activity against *P. falciparum* (MLD_50_/IC_50_).

### Continuous cultures of *P. falciparum*

CQ-R *P. falciparum* parasites (W2 clone) [25] and CQ-S (3D7 strain), originally received from New York University Medical School, were maintained in continuous culture at 37°C in human erythrocytes (A^+^) using complete medium (RPMI 1640 supplemented with 10% blood group A^+^ human serum) changed daily [26]. Before tests with the molecules were performed, ring stage parasites were synchronized using sorbitol [27] and the parasitaemia and haematocrit were adjusted; 180 μL/well was added to 96-well microtiter plates (Corning, Santa Clara, CA, USA) containing the diluted compounds tested in triplicate. Drug activity was evaluated in relation to control cultures with no drugs [28] and was measured using the anti-histidine-rich protein II (HRPII) test as described [29]. CQ was used in each test as a control, and two to four experiments were performed for each parasite strain.

### *In vitro*tests of drug activity

The anti-HRPII test was performed using *P. falciparum* cultures adjusted for 0.05% parasitaemia and 1.5% haematocrit. The cultures were added to plates containing the diluted drugs, followed by incubation for 24 h under standard culture conditions [26]. The contents of six wells with no drugs were harvested in each test plate, pooled in microtubes, and frozen for later use to measure the background parasite growth as described [29]. After a 48 h incubation, the plates were frozen and thawed twice, and 100 μL of lysed cells from each well was added to a plate that had been precoated overnight at 4°C with a primary anti-HRPII monoclonal antibody (MPFM-55A ICLLAB®, USA) for an immunoassay. Phosphate-buffered saline (PBS) containing 0.05% Tween and 4% bovine serum albumin was used for plate blocking (3 h at room temperature) to avoid nonspecific antibody binding. After 1 h at room temperature, the plate was washed, and 100 μL/well of a secondary antibody solution (MPFG-55P ICLLAB®, USA) was added, followed by incubation with 3,3′,5,5′-tetramethylbenzidine (TMB) chromogen (KPL, Gaithersburg, MD, USA) in the dark. The reaction was stopped using 1 M sulfuric acid, and the absorbance was read (450 nm) in a spectrophotometer (SpectraMax340PC^384^, Molecular Devices). The anti-*P. falciparum* drug activity was evaluated by comparing the parasite growth in the drug-free control cultures, considered 100% growth, with that in the drug test cultures. Using curve-fitting software (Microcal Origin Software 5.0, Inc.), a sigmoidal dose–response curve was generated, enabling the determination of drug IC_50_ values. CQAns presenting IC_50_ ≤ 1 μM were considered active; between 1–5 μM as partially active; and above 5 μM as inactive.

### Protocol for animal use in the anti-malarial tests

The protocol for animal use was approved by the Ethics Committee for Animal Use (CEUA LW-23/13) of the Oswaldo Cruz Foundation (Fiocruz). Only Swiss adult female mice (20 ± 2 g weight) were used; they were raised at the animal facilities at FIOCRUZ-Minas.

### Anti-malarial tests against *P. berghei*in mice

The suppressive test was performed as described [30] using the *P. berghei* NK65 blood parasites, with some modifications [31]. Briefly, the blood parasites were maintained through weekly blood passages in mice. For the experiments, groups of 20–30 mice were inoculated with 1 × 10^5^ infected erythrocytes. Approximately three hours later, they were randomly distributed into groups of five to six mice per cage, which were treated daily by gavage for four consecutive days. All the compounds were freshly diluted in 3% DMSO (Sigma-Aldrich) in RPMI medium and were administered orally at doses of 25 mg/kg or 50 mg/kg. The control mice received the drug vehicle. On days 5 and 7 after parasite inoculation, blood was taken from the tail of each mouse and used to prepare thin smears, which were methanol-fixed, Giemsa-stained and examined microscopically to determine parasitaemia. The inhibition of parasite growth was evaluated in relation to parasitaemia in the untreated mice, which were considered to have 100% parasite growth.

A *P. berghei* NK65 CQ-resistant strain maintained at -70°C, selected by increasing doses of CQ, maintained in mice under constant drug pressure (CQ 150 mg/kg) [32] and stored at -70°C was also defrosted and used to inoculate mice. When parasitaemic, the drug-resistant parasites were subjected to serial blood passages maintained under CQ treatment with increasingly higher doses, up to 150 mg/kg, then used for the experiments. The mice were inoculated with 10^7^ parasitized RBCs using blood parasites from a donor with parasites resistant to 150 mg/kg of CQ. Groups of five mice each were separated 24 h after the parasite inoculation, then treated orally with the test CQAn at doses of 25 or 50 mg/kg for three consecutive days. Two control groups were used in each experiment; one received CQ at 150 mg/kg and the other was infected but treated with vehicle only (no drugs). The inhibition of parasitaemia was evaluated as described for the mice inoculated with the CQ-sensitive parasites. A compound was considered active when it inhibited parasitaemia more than 40% in relation to the infected but vehicle-treated (no drugs) group.

### Inhibition of β-haematin formation assay

The assay was performed using a lipid as a catalyst to promote crystallization [33]. Briefly, drug stock solutions were prepared in DMSO and were used at a final concentration of up to 30 mM. A haem stock (10 mM) was made in DMSO and was diluted to 50 μM with 100 mM sodium acetate (pH 4.8). A 10 mM 1-monooleoyl-rac-glycerol (MOG) stock was made in ethanol and was sonicated before being added to a 50 μM haem stock to make 25 μM MOG–50 μM haem in 100 mM sodium acetate (pH 4.8). The 25 μM MOG–50 μM haem solution was sonicated and added to the assay plate at 100 μL/well. The plates were incubated at 37°C for 2 h to allow crystallization, followed by the addition of 100 μl of 200 mM sodium bicarbonate (pH 9.1) to solubilize any remaining monomeric haem. After incubation for 30 min at room temperature, the amount of solubilized monomeric haem was determined by measuring the absorbance at 405 nm. Finally, 20 μl of 1 M sodium hydroxide was added to the plates to dissolve any crystals that had been formed, and the absorbance was read at 405 nm to determine the total amount of haem present in each well. The inhibition of haem crystallization was determined as a function of the amount of monomeric haem that was not crystallized divided by the total amount of haem present in the assay mixture. The results are expressed as IC_50_ values based on the percentage inhibition of β-haematin formation by the CQAn.

### Docking studies

The structures of the inhibitors studied were constructed and optimized with the program Spartan 08 [34] using the Merck Molecular Force Field (MMFF) method [35]. In addition, the partial charges were calculated by single-point energy calculations using the RM1 method [36]. The crystallographic structures of *Pf*LDH and *Hss*LDH were downloaded from the Protein Data Bank [37] under the codes 1LDG (resolution 1.74 Å and R factor = 0.197) and 1IOZ (resolution 2.10 Å and R factor = 0.179), respectively.

The program Molegro Virtual Docker® (MVD) [38] was used for the docking studies, and the methodology used was validated by re-docking. To observe possible interactions of the ligands with the solvent present in the crystal, 35 water molecules from a cavity approximately 10 Å in size were considered for the calculations with *Pf*LDH, whereas 54 molecules were considered for the calculations with *Hss*LDH. The docking region was restricted to a radius of 15 Å from the centre of the cavity inside each enzyme. The best ligand conformations inside the enzymes were chosen based on 100 runs for each ligand, and considering the superposition to the structure of NADH molecules present in the crystallographic structures combined with the best values for interaction energy, and for the H-bonds with water molecules and the residues of the active sites.

The volumes of the cavities of the NADH-binding sites inside *Pf*LDH and *Hss*LDH were determined using MVD® [38].

## Results

### Anti-*P. falciparum*activity and selectivity indexes of CQAns

All the CQAns were active *in vitro* against CQ-R *P. falciparum* parasites, exhibiting half-maximal inhibitory concentration (IC_50_) values below 1 μM; the most active molecule was CQAn33 (IC_50_ = 0.02 ± 0.001 μM). Tested against the CQ-S parasites in parallel, CQAn34 and CQAn37 were active; CQAn33, CQAn39 and CQAn45 were partially active; and CQAn28 was inactive (Table 1).

**Table 1:** **Activity of CQ and CQAn against** ***P*** **.** ***falciparum*** **(IC**_**50**_ **) CQ-R (W2) or CQ-S (3D7) parasites**

Molecule	***Chemical structure***	***Anti***- ***P***. ***falciparum***activity (IC _50_in μM)*	
		***W2***( ***CQ***- ***R***)	***3D7l***( ***CQ***- ***S***)
CQAn28		0.71 ± 0.03	14.87 ± 3.58
CQAn33		0.02 ± 0.001	1.17 ± 0.10
CQAn34		0.23 ± 0.15	0.3 ± 0.01
CQAn37		0.07 ± 0.03	0.57 ± 0.24
CQAn39		0.64 ± 0.29	2.39 ± 0.11
CQAn45		0.77 ± 0.21	1.17 ± 0.00
Chloroquine		0.38±0.03	0.081 ± 0.019

*Average ± SD of the IC_50_ is based in two to four experiments performed with each parasite strain using anti-HRPII method.

Most of the CQAns were less toxic than CQ (MLD_50_ = 410.4±26.6 μM), with MLD_50_ values ranging from 943 to 1699 μM. The ratios of cytotoxicity to activity (selectivity index, SI) of compounds CQAn37, CQAn33, CQAn39, and CQAn34 were 24275, 7645, 5011, and 4101, respectively, considering the CQ-R parasites. For CQAn28 and CQAn45, the SI values were 2125 and 119, respectively; and for CQ, the SI was 1080. Using CQ-S *P. falciparum* parasites, none of the CQAns showed an SI higher than CQ (SI = 5679). The highest SI values were exhibited by CQAn34 (SI = 3144) and CQAn37 (SI = 2981); all the other CQAns had SI values below 1500 (Table 2).

**Table 2:** **Cytotoxicity of CQ and CQAn and SI on** ***P*** **.** ***falciparum*** **parasites and therapeutic activity or selectivity index (SI), a ratio between toxicity and activity**

Molecule	Cytotoxicity* ***MLD***_***50***_(μM)	***SI***(***MLD***_***50***_/***IC***_***50***_)	
	HepG2 cells	W2 (CQ-R)	3D7 (CQ-S)
CQAn28	1508.56 ± 344.50	2125	101
CQAn33	152.89 ± 12.86	7645	131
CQAn34	943.29 ± 73.15	4101	3144
CQAn37	1699.24 ± 10.07	24275	2981
CQAn39	≥3207.08	5011	1342
CQAn45	1015.84 ± 42.97	1319	868
Chloroquine	410.4±26.6	1080	5,679

*Average ± SD of two to four experiments measured through MTT-colorimetric assay.

### *Anti*-*P. berghei*malaria activity of the CQAns

All the compounds (CQAn28, CQAn33, CQAn34, CQAn37, CQAn39, and CQAn45) that were tested in mice with *P. berghei* caused between 73% and 100% inhibition of parasitaemia after treatment with a 25 mg/kg oral dose, compared with the untreated control group on day 5. On day 7, the parasitaemia inhibition reached 93% (Table 3). CQ, used as a control, inhibited parasitaemia by 100% and 97% on days 5 and 7, respectively. The 50 mg/kg dose of CQAn33, CQAn37 and CQ caused 100% inhibition of *P. berghei* parasitaemia on days 5 and 7 after infection. Two of the five mice receiving CQAn37 and all the mice receiving CQ survived the infection at least 30 days after the end of treatment; CQ cured the mice.

**Table 3:** **Anti-malarial activity of chloroquine (CQ) and its analogs (CQAn) against** ***P*** **.** ***berghei*** evaluated as percentage reduction of parasitaemia on days after inoculation with either a CQ sensitive or resistant strain of the parasite*

CQAn	CQ-Sensitive 25 mg/Kg		CQ-Resistant 50 mg/Kg	
	Day 5	Day 7	Day 5	Day 7
**28**	94^a^	24^a^	0^b^	0
**33**	73^a^	57^a^	53	52
**34**	99	81	0^b^	0
**37**	100	93	47	33
**39**	95	92	62	30
**45**	88	50	63	51
**CQ**	100	97	0^c^	0^c^

*Reduction calculated in relation to control non-treated mice (100% of parasite growth).

^a^Adapted from de Souza et al. [19]; all the compounds were used at 50mg/Kg, except as indicated, in the dose of 25 mg/Kg because there was not enough material for a higher dose^b^. In the case of CQ used as control in the test with the resistant parasites, the dose used was 150 mg/Kg, which was totally inactive, as expected^c^.

The CQAns were evaluated against a CQ-resistant *P. berghei* strain (maintained by constant drug pressure at a dose of 150 mg/kg daily until the last passage before the test). The dose of 50 mg/kg was active for all the CQAns on day 5. CQAn45 caused the highest parasitaemia reduction (63%), followed by CQAn39, CQAn33 and CQAn37, which reduced parasitaemia by 62%, 53% and 47%, respectively. CQAn28 and CQAn34 were inactive at 25 mg/kg. As expected, CQ at the highest tolerated dose of 150 mg/kg was inactive against the CQ-resistant *P. berghei* parasites in mice (Table 3).

### Inhibition of β-haematin formation by CQAn

The inhibition of β-haematin formation was evaluated for CQAn33 and CQAn37, the most active CQAns against *P. falciparum in vitro*. The results showed that CQAn33 inhibited β-haematin formation with an IC_50_ value similar to that of CQ, whereas CQAn37 inhibited β-haematin formation at a concentration 2-fold higher than that of CQ (Table 4).

**Table 4:** Inhibitory concentrations of β-haematin formation by chloroquine (CQ) and two analogs (CQAn)*

CQAn	Exp			Mean ± SD
	1	2	3	
**33**	4.7	3.0	5.6	4.3 ± 1.3
**37**	2.7	10.2	7.5	8.9 ± 3.8
**CQ**	0.6	2.0	5.0	3.5 ± 2.2

*Results expressed as IC_50_ in mM.

### Docking studies and the mechanism of activity of the CQAns

The root-mean-square deviation (RMSD) values obtained through re-docking of the NADH structure inside each crystal were lower than 2.00 Å in the case of *Pf*LDH and *Hss*LDH (Tables 5 and 6). The cavities of the NADH-binding sites inside *Pf*LDH and the human lactate dehydrogenase enzyme (*Hss*LDH) were determined to have volumes of 117,248 and 7,936 Å^3^, respectively. Both cavities were generated around the NADH structure present inside the crystallographic structures. The results suggest that the new CQAns studied have affinities for the NADH-binding sites of *Pf*LDH and *Hss*LDH. These compounds were able to dock and to establish highly stable H-bonds with amino acids and water molecules in the sites, as reflected by the highly negative total intermolecular energy (IE) values (Tables 5 and 6). The IE values were less negative inside *Hss*LDH than inside *Pf*LDH.

**Table 5:** **Docking results of NADH, CQ and CQAn inside** ***Pf*** **LDH**

Ligand	Intermolecular energy (kcal mol ^-1^)	H-bond energy (kcal mol ^-1^)	Residues and water molecules involved in H-bonds	Distance (Å)	H-bond energy (kcal mol ^-1^)
NADH RMSD 1.437	-180.166	-11.878	Met30(2)	3.293	-0.331
				2.972	-1.534
			Gly99	3.329	-1.355
			Asp53	3.259	-1.707
			Ile31	2.267	0.288
			Gly29	2.876	-0.758
			Gly32	3.301	-1.495
			Phe100	3.213	-1.933
			Ser245	3.585	-0.077
			Leu163(2)	3.536	-0.060
				3.522	-0.392
			His195	3.595	-0.025
			Val138	2.737	-2.500
			H_2_O (2)	3.466	-0.671
				3.172	-2.142
			H_2_O	1.004	11.271
			H_2_O	2.946	-2.500
			H_2_O	1.992	2.675
			H_2_O	3.111	-2.445
			H_2_O	3.086	-2.500
			H_2_O	2.911	-2.500
CQ	-148.459	-3.232	Ile31	3.344	-1.278
			Met30	3.013	-0.962
	(-133.455)		Asn140	2.866	-0.992
			H_2_O	3.210	-1.951
CQAn28	-131.266	-4.086	Asp53	3.283	-1.586
	(-123.897)		Gly99	3.014	-2.500
CQAn33	-135.340	-4.602	Val138	2.593	-2.105
			Thr97	2.843	-2.498
	(-123.651)		H_2_O	3.093	-2.500
			H_2_O	2.758	-2.500
			H_2_O	3.336	-1.321
CQAn34	-123.505	-3.088	Asp53	2.762	-1.575
	(-115.999)		Thr97	3.127	-1.513
			H_2_O	2.967	-2.500
CQAn37	-141.264	-2.500	Asp53	2.938	-2.500
	(-131.154)		H_2_O	3.210	-1.951
CQAn39	-145.727	-2.500	Asp53	2.869	-2.500
	(-123.226)		H_2_O	3.263	-1.685
CQAn45	-131.123	-1.544	Gly99	2.999	-1.544
	(-110.764)

**Table 6:** **Docking results of NADH, CQ and CQAn inside** ***Hss*** **LDH**

Ligand	Intermolecular energy (kcal mol ^-1^)	H-bond energy (kcal mol ^-1^)	Residues and water molecules involved in H-bonds	Distance (Å)	H-bond energy (kcal mol ^-1^)
NADH RMSD 0.956	-191.613	-20.561	Asp119 (2)	3.085	-2.500
				2.817	-1.588
			Ser145	3.131	-2.343
			Ala146	2.627	-0.675
			Lys147	2.635	-2.500
			Lys117	3.482	-0.592
			Gly15	2.566	-2.215
			Val14	2.851	-0.277
			Lys16 (2)	2.940	-2.500
				2.470	-0.726
	(-190.630)		Gly13	2.975	-0.360
			Ala18	3.288	-1.561
			Ser17(2)	2.794	-1.529
				3.315	-1.193
			H_2_O	2.725	-2.500
			H_2_O (2)	3.132	-2.342
				2.578	-2.313
			H_2_O (3)	3.359	-1.203
				2.982	-2.500
				3.213	-1.934
CQ	-127.669	-2.983	Ala146	3.390	-0.833
	(-104.576)		Asn116	2.785	-2.149
			H_2_O	3.438	-0.812
CQAn28	-123.535	-3.669	Ala146	3.218	-1.394
	(-114.612)		Asp116	2.922	-2.275
			H_2_O	3.193	-2.033
CQAn33	-125.365	-3.298	Lys147	3.551	-0.143
	(-112.274)		Ala146	3.139	-1.599
			Asn116	3.206	-1.556
CQAn34	-113.935	-3.842	Asn116	2.974	-2.074
	(-104.054)		Ala146	3.126	-1.768
			H_2_O	3.204	-1.979
CQAn37	-121.066	-3.588	Gly15	3.398	-0.163
			Asn116	3.166	-1.425
	(-110.364)		Ala146	3.051	-1.904
			Lys147	3.573	-0.010
CQAn39	-129.262	-2.498	Asn116	2.799	-2.068
	(-107.522)		Ala146	3.493	-0.430
CQAn45	-128.632	-1.411	Asn116	3.401	-0.843
	(-94.840)		Ala146	3.420	-0.569
			H_2_O	3.074	-2.500

Inside *Pf*LDH, all the compounds (except CQAn45) H-bonded to Asp53 and/or Thr97 plus water molecules and displayed good (negative) H-bond energy values. The intermolecular energies were ranked as follows: CQ > CQAn39 > CQAn37 > CQAn33 > CQAn28 > CQAn45 > CQAn34, with lower values than NADH. The highest H-bond energy was observed for CQAn33 and the lowest for CQAn45.

Most of the active-site residues of *Pf*LDH involved in H-bonds differed from those of *Hss*LDH. Thus, the residues of *Pf*LDH involved in H-bonds with CQAn33 and CQAn37 were Thr97, Val138 and H_2_0; and Asp53 and H_2_0, respectively. The residues of *Hss*LDH for the same molecules were Lys147, Ala146 and Asn116; and Gly15, Asn116, Ala146 and Lys147, respectively.

**Figure 2:**
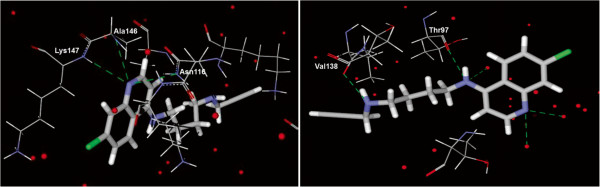
**Interactions of CQAn33 inside HssLDH (left) and PfLDH (right).** Water molecules inside the active sites are represented as red spheres and H-bonds are shown as dotted green lines.

**Figure 3:**
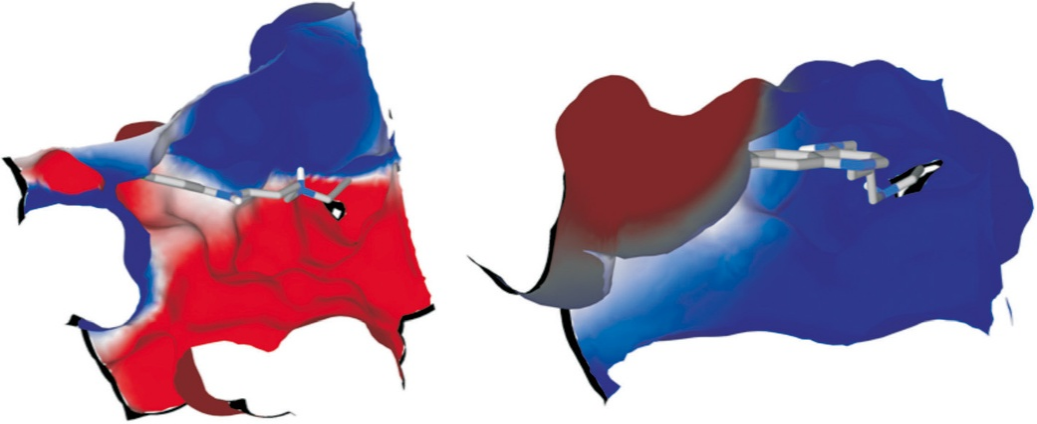
**Surface representation of the binding sites of CQAn33 (NADH pockets) inside HssLDH (left) and PfLDH (right).** Red means negative charges, blue means positive charges and white means neutral.

To better understand the experimentally observed selectivity, the electrostatic potentials of the NADH-binding sites inside *Pf*LDH and *Hss*LDH were determined. The results showed differences in the binding pockets: inside the *Pf*LDH pocket, the electrostatic potential was totally positive, whereas inside *Hss*LDH, it was mostly negative, as illustrated by CQAn33 docked inside both enzymes (Figures 2 and 3).

## Discussion

Although CQ-R is widespread, CQAns may help to overcome the drug resistance, especially considering that it is believed to be stage specific [39] and/or related to the compound structure [15]. Indeed, various CQAns have shown potent antiplasmodial activity against CQ-R *P. falciparum* blood parasites [40–42]. Among the six CQAns tested against *P. falciparum* in the present work, all exhibited high activity against CQ-R parasites. CQAn28, 33, 37 and 39 were more potent against the CQ-R W2 clone than against the 3D7 CQ-S parasites.

Variation in side chains seems to be a promising strategy concerning the anti-malarial activity of CQAns, and small changes in the spacer length can dramatically affect the activity exhibited against CQ-S and CQ-R parasites [43]. Moreover, the presence of a terminal alkyne seems important: CQAn33, which has a terminal alkyne, showed an IC_50_ more than 10-fold higher than that of CQ, which has no terminal alkyne.

The activity of 4-aminoquinolines involves their binding to haematin in the monomeric and dimeric forms, inhibiting haemozoin formation and resulting in parasite death [44]. The activity of CQ against *P. falciparum* also involves interactions with the parasite enzyme *Pf*LDH [20], as shown by *in silico* studies with docking and molecular dynamics in the present work. The complex formed between the dimeric haematin and the quinolinic drugs inhibited *Pf*LDH, as shown with other quinoline derivatives [21].

In previous studies, RMSD values below 2.00 Å were obtained for the plasmodial and human enzymes in re-docking studies, which validates this methodology [45–47]. The intermolecular energy values for the CQAns were higher than for CQ and NADH, showing that the molecules studied here are weak inhibitors of *Pf*LDH and suggesting that they have a different mechanism of action.

CQAn33 and CQAn37, the molecules with the highest SI *in vitro*, were also evaluated for their ability to inhibit β-haematin formation. The results showed that CQAn33 inhibited β-haematin formation with an IC_50_ similar to that of CQ, whereas CQAn37 exhibited an IC_50_ more than 2-fold higher than CQ, which is in accordance with this molecule being less active than CQAn33 against *P. falciparum*. The results obtained for anti-*P. falciparum* activity through the HRPII test showed a rather higher activity of these CQAns in relation to CQ, suggesting another mechanism of action than that related to haemozoin formation, such as apoptosis, autophagy or the inhibition of P-glycoprotein-mediated transport. Metacaspase-like proteins, which are related to caspases [48], have previously been shown to be inducers of apoptosis in *P. falciparum* parasites exposed to CQ, with more intense DNA fragmentation in CQ-R parasites than in CQ-S [49]. Autophagy-related proteins have been identified in *P. falciparum* [50, 51]. Indeed, after CQ pressure, *P. falciparum* parasites exhibited cytoplasmic vacuolization [52], a sign of autophagic cell death [53] rather than of apoptosis. A recent study showed that CQ significantly inhibited P-glycoprotein-mediated transport [54], which is involved in drug evasion in *P. falciparum* [55].

The highest and lowest H-bond energies were observed for CQAn33 and CQAn45, respectively, a result that corroborates the *in vitro* data showing that CQAn33 was the most active molecule and CQAn45 the least active against *P. falciparum*. Another difference is the residues of the plasmodial and human enzymes involved in the H-bonds, specific for CQAn33 and CQAn37; this result is consistent with them having the highest SIs.

The differences in the binding pockets of the studied compounds’ tails inside *Pf*LDH and *Hss*LDH could be explained by a large concentration of negative charges due to the presence of the alkyne groups. This finding suggests a better stabilization inside the more positive pocket of *Pf*LDH, which is caused by the size of the cavity and increases drug specificity. Indeed, the smaller cavity of *Pf*LDH allows for better accommodation of the ligands than inside *Hss*LDH.

The most active CQAn *in vitro* also inhibited *P. berghei* parasitaemia in mice, as shown in the present work and in our previous studies with the same compounds [19]. An increase in mouse survival resulting from treatment with CQAn37 may well be a consequence of the alkyl side chain altering the structure-activity relationship in the terminal nitrogen [15], which may favor drug lipophilicity. All the CQAns inhibited CQ-R *P. berghei* parasitaemia in mice, translating the *in vitro* data, with the exception of CQAn28 and 34, which is likely a result of metabolism [13] or of the structure-activity relationships inherent to the resistance phenotype, e.g., those related to *pfcrt* [7, 56], also *in vivo* [57].

The data reported herein highlight the use of CQAns in malaria-endemic areas where drug resistance has been reverted, i.e., sensitivity to CQ has reappeared [58]. CQ is the most widely used anti-malarial drug worldwide [2] due to its low cost, high efficacy and lack of toxicity. In addition, the cost of a treatment course is rather low at US$0.21 per patient [59]. The CQAns evaluated in this study are generated in two steps using readily available, inexpensive starting materials and can be produced in good yields [22] at a cost expected to be as low as that of CQ. Thus, the CQAns studied in the present work may be produced at large scale to proceed in drug development for anti-malarials, since they are promising alternatives for malaria control.

## Conclusions

Among the six CQAns tested *in vitro*, three were more active than CQ, inhibiting 50% of *P. falciparum* growth at low doses: CQAn33 (IC_50_ 0.02 ± 0.001 μM), CQAn34 (IC_50_ 0.23 ± 0.15 μM), and CQAn37 (IC_50_ 0.07 ± 0.03 μM). All were more active against the CQ-R (W2) parasites than against the CQ-S (3D7) parasites, which suggests the absence of cross-CQ-R. In addition, the therapeutic indexes (or SI) of all CQAns, with the exception of CQAn45, were higher than that of CQ. Docking studies with CQAn33 and CQAn37 corroborated the outstanding anti-*P. falciparum* activity observed herein and showed that CQAn33 had the highest H-bond energy with the plasmodial enzyme, indicating specificity for *Pf*LDH. Additionally, they showed that the CQAns are weak inhibitors of *Pf*LDH, suggesting a different mechanism of action. The inhibition of β-haematin formation by CQAn33 and CQAn37 also suggested mechanisms of action other than those related to haemozoin formation. The *in vivo* results with CQAn34, CQAn37, CQAn39, and CQAn45 confirm previous data showing their high anti-malarial activity. Furthermore, the CQAns also inhibited the parasitaemia caused by CQ-R *P. berghei* but CQ did not, translating *in vitro* to *in vivo* data. However, CQAn28 and CQAn34 did not inhibit CQ-R *P. berghei* parasitaemia, what may be a result of metabolism issues or structure-activity relationships related to the resistant phenotype. Altogether, the present results highlight the CQAns studied here as promising anti-malarial agents due to their SI and *in vivo* activity against malaria in mice.
